# miRNA biomarkers in renal disease

**DOI:** 10.1007/s11255-021-02922-7

**Published:** 2021-07-06

**Authors:** Beata Franczyk, Anna Gluba-Brzózka, Robert Olszewski, Magdalena Parolczyk, Magdalena Rysz-Górzyńska, Jacek Rysz

**Affiliations:** 1 Department of Nephrology, Hypertension and Family Medicine, Medical University of Lodz, Lodz, Poland; 2 Department of Gerontology, Public Health and Education, National Institute of Geriatrics Rheumatology and Rehabilitation, Warsaw, Poland; 3 Department of Ultrasound, Institute of Fundamental Technological Research, Polish Academy of Sciences, Warsaw, Poland; 4 Department of Ophthalmology and Visual Rehabilitation, Medical University of Lodz, Lodz, Poland

**Keywords:** Chronic kidney disease, miRNA, Biomarkers

## Abstract

Chronic kidney disease (CKD), which is characterized by the gradual loss of kidney function, is a growing worldwide problem due to CKD-related morbidity and mortality. There are no reliable and early biomarkers enabling the monitoring, the stratification of CKD progression and the estimation of the risk of CKD-related complications, and therefore, the search for such molecules is still going on. Numerous studies have provided evidence that miRNAs are potentially important particles in the CKD field. Studies indicate that some miRNA levels can be increased in patients with CKD stages III–V and hemodialysis and decreased in renal transplant recipients (miR-143, miR-145 and miR-223) as well as elevated in patients with CKD stages III–V, decreased in hemodialysis patients and even more markedly decreased in renal transplant recipients (miR-126 and miR-155). miRNA have great potential of being sensitive and specific biomarkers in kidney diseases as they are tissue specific and stable in various biological materials. Some promising non-invasive miRNA biomarkers have already been recognized in renal disease with the potential to enhance diagnostic accuracy, predict prognosis and monitor the course of disease. However, large-scale clinical trials enrolling heterogeneous patients are required to evaluate the clinical value of miRNAs.

## Introduction

Chronic kidney disease (CKD), which is characterized by the gradual loss of kidney function, is a growing worldwide problem nowadays due to CKD-related morbidity and mortality. In 2017, the general prevalence of CKD was estimated to be 9.1% (95% UI 8.5–9.8) in the world’s population, while 5.0% (4.5–5.5) of population was suffering from CKD stages 1–2, 3.9% (3.5–4.3) from stage 3, 0.16% (0.13–0.19) from stage 4, 0.07% (0.06–0.08) from stage 5 for and 0.041% (0.037–0.044) was undergoing dialysis [1]. The presence of CKD was associated with 1.2 million (95% uncertainty interval (UI) 1.2–1.3) deaths in 2017, while subsequent 1.4 million (1.2–1.6) died from cardiovascular disease that was attributable to impaired kidney function. According to estimations, over 2.5 million of people undergo renal replacement therapy, and this amount is projected to double (5.4 million) by 2030 [2]. The enhanced cardiovascular morbidity and mortality in the course of CKD is associated with the reduction in the number of functional nephrons which leads to the accumulation of uremic toxins, such as p-cresyl sulfate (PCS) and indole-3-acetic acid (IAA) [3, 4]. Moreover, CKD is associated with mineral and bone metabolism disorders resulting in vascular calcifications which are the key factor leading to the aggravation of renal impairment and atherosclerosis [5].

Due to the fact that there are no reliable and early biomarkers enabling the monitoring, the stratification of CKD progression and the estimation of the risk of CKD-related complications the search for such molecules is still going on. Numerous studies have provided evidence that miRNAs are potentially important particles in the CKD field since some of them seem to be involved in kidney diseases. Altered expression of miRNAs has been shown to be involved in the initiation and the progression of numerous pathologic processes, such as diabetic nephropathy, renal cancer and renal injury [6]. Moreover, serum miRNAs are highly stable in blood and they are suggested to be diagnostic and prognostic biomarkers for numerous diseases [7]. The relationship between miRNA levels and CKD has been found in cellular and animal models [8–10], but also in the study of humans (though only limited data concerning miRNA levels in human diseases are available).

## miRNAs

The processes of gene regulation involves the participation of newly discovered class of particles, which could be divided, on the basis of their size, into short noncoding RNA (< 200 nucleotides including miRNA, snoRNA, and piRNA) or long noncoding RNA (lncRNA and circular RNAs, > 200 nucleotides) [11, 12]. However, in this review, we will focus on the miRNAs only. Currently, there are approx. 2000 discovered miRNA, and their encoding genes occupy approximately 3% of the genome [11, 13, 14]. These particles are endogenous, small (approx. 20–25 nucleotides) noncoding RNAs which act as negative regulators of gene expression, since they are responsible for the degradation or translational inhibition of their target mRNAs [15]. MiRNAs are initially transcribed by RNA polymerase II in the form of longer RNA products (called Pri-miRNA), which are subsequently cleaved by RNase III (Drosha) and DiGeorge syndrome critical region 8 (DGCR8) in the nucleus which results in miRNA maturation. Formed pre-miRNA hairpins with length of approx. 60–70 nt are exported by exportin into cytoplasm, where they are cleaved by the Dicer RNase III into a double-stranded miRNA/miRNA duplex (approx. 22 bp). In the final step, one of strands is incorporated in RISC-complex (RNA-induced-silencing complex), which carries it to target mRNA (messenger RNA) to silence gene expression, while the second strand is either rapidly degraded or becomes miRNA with distinct biological effects [11].

Sequences of the seed region of mature miRNA are conserved, however, outside this region several mismatches are common, and therefore, single miRNA is able to modulate the expression of multiple target genes via the inhibition of target mRNA translation, or the promotion of their degradation which affects numerous levels of developmental pathways [11]. During the translation stage, miRNAs can inhibit the initiation and elongation steps of a given protein to diminish its expression [16–18]. Moreover, they can enable the sequestering of targeted mRNAs to processing bodies in cytoplasm for degradation [18–20]. Finally, they are involved in the transcriptional gene silencing by targeting the promoter region [18, 21]. The presence of perfect or imperfect complementarity between miRNA and its target mRNA enables the regulation of multiple genes. miRNA-related gene expression can result in irreversible activation of signaling pathways, it simplifies the switch of cellular phenotype under pathological conditions as well as promotes disease progression [6]. Mitchell et al. [22] for the first time demonstrated that miRNAs are present in human plasma and that they are stable there despite known high RNase activity of plasma. The activity of miRNAs can be modulated by long noncoding RNAs (lncRNAs) which can hinder the function of specific miRNAs by acting via ‘sponge-like’ effects or exert effects via chromatin remodeling [13]. According to some studies, the interactions between these two types of noncoding RNAs are involved in the pathogenesis and progression of various diseases [13, 15].

**Fig. 1:**
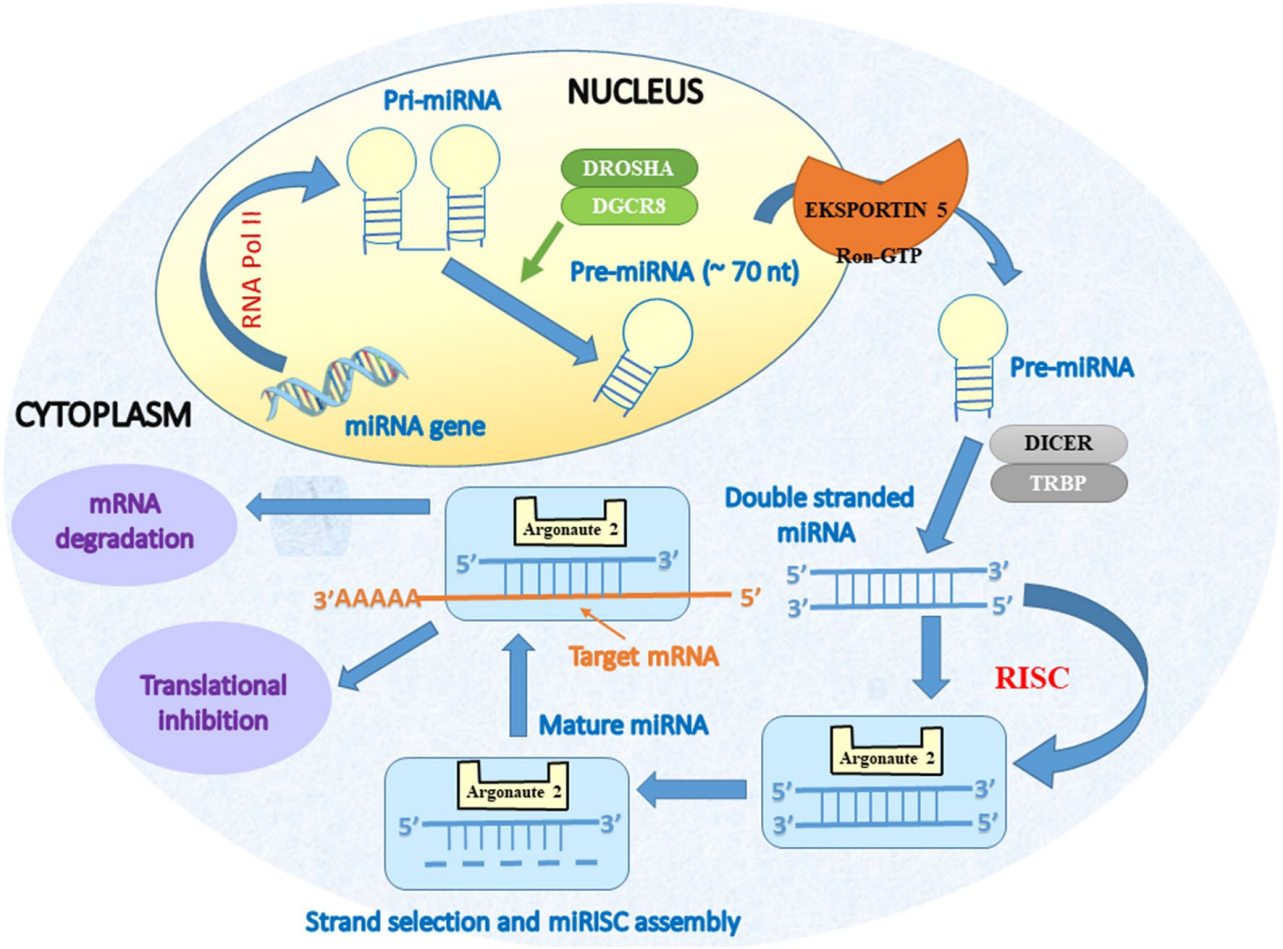
miRNA biogenesis and mechanism of action

The summary of miRNA biogenesis and mechanism of action is presented on Fig. 1

Recently, miRNAs have attracted interest as potential biomarkers enabling the detection of some diseases, the assessment of their severity and the monitoring of the course.

## The pros and cons of miRNA as biomarkers

Currently, there are no biomarkers in the field of nephrology, which could be considered as ideal, i.e., non-invasive, reliable, and having a high sensitivity and a high specificity [23]. miRNAs might meet the specificity, sensitivity, and reproducibility criteria that are required for a reliable, non-invasive biomarker provided that normalization strategies for accurate quantification and appropriate controls are utilized. Due to the fact that miRNA can regulate molecular pathways and their expression can be disease-specific, it seems that these molecules offer the opportunity to distinguish between similar disease states [24]. For example, serum miR-148b and miR-let-7b levels were reported to discriminate patients with IgA nephropathy from both controls and patients with other forms of glomerulonephritis in a large international study [25]. The profiling of MiRNAs can serve also as relevant biomarker also in the processes of epithelial–mesenchymal transition (EMT), fibrosis, inflammation, and the activation of renal stem cells, which may be involved in the progression of kidney disease [26–28].

However, the fact that one miRNA can be possibly involved in several different diseases remains a problem. Moreover, the normalization of miRNA expression levels during the analyses remains a problem [29]. Real-time PCR is suitable for the analysis of miRNA levels, as this technique is easy and accurate, however, the determination of appropriate endogenous circulating miRNAs which will enable the normalization of expression is not an easy task. U6 and miR-1249 have been used for some time until their high variability was found [29]. Standard utilization of miRNA signature for the diagnostic or prognosis of CKD still requires a lot of research and the introduction of uniform method of miRNAs’ expression levels quantification. So far, researchers are using various techniques which is associated with poor reproducibility of obtained results [11, 29]. Due to the fact that the concentration of miRNA in blood is low, standardization of analysis if of key importance. To avoid experimental bias, the use of synthetic exogenous miRNA, such as C. elegans miR-39, has been proposed [29]. It seems also that the time required for the analysis of miRNA with the use of RT-PCR is too long for the requirements of clinical use. Therefore, alternative techniques, including those using quantum dot-based Förster resonance energy transfer or electrochemical detection of miRNAs in urine samples have been suggested [30, 31].

Another advantage of circulating miRNAs as biomarkers is their stability in human plasma and serum, which is believed to be associated with their containment in exosomes (cell-derived microvesicles) where they are protected from circulating ribonucleases and their binding to Argonaut proteins and HDL [32]. Exosomes are released from most types of cells in various physiological and pathological states [33]. Moreover, their attractiveness as biomarkers is also associated with the tissue‐specific nature of miRNA expression [34, 35]. miRNAs are present in most solid tissues and they exhibit great stability in formalin-fixed samples [36–38].

miRNAs can be used not only in the diagnosis, but also as predictive markers [24]. For example, the upregulation of urinary (and to a lesser extent plasma) miR-21 has been demonstrated to predict AKI progression in patients undergoing cardiac surgery [39]. Moreover, miRNAs were demonstrated to predict progression in chronic kidney disease (CKD).

## miRNA in CKD development and progression

The contribution of miRNAs to chronic kidney disease progression involves their regulation of mRNAs participating in renal homeostasis [40]. However, their impact on clinical outcome remains poorly understood [40]. Studies of animal models have demonstrated the involvement of miRNA in the progression of glomerular and tubular damage [41, 42]. There are also some reports of the role miRNA in IgA nephropathy (intrarenal expression of miR‐200c, miR‐141, miR‐205 and miR‐192 correlated with disease severity and progression), lupus nephritis (differential expression of 16 miRNAs in peripheral blood mononuclear cells), renal cancers (more accurate tumor classification than mRNA analysis), however, there are just few studies enrolling humans, which analyzed miRNA expression in chronic kidney disease [43–45]. Some miRNAs have been demonstrated to be tissue‐restricted and expressed specifically in the kidney [46]. The expression of miR‐192, miR‐194, miR‐204, miR‐215 and miR‐216 are found to be higher in kidney than in other organs [35]. In turn, low levels (compared to other tissues) or the absence of a given miRNAs in the kidney was suggested to enable the renal specific expression of target proteins which are vital for kidney function [27, 35, 46]. The study of miRNA and mRNA expression profiling on renal biopsy sections revealed that miR-30d, miR-140-3p, miR-532-3p, miR-194, miR-190, miR-204, and miR-206 were downregulated in progressive cases and they upregulated 29 target mRNAs participating in intracellular signaling, inflammatory response, apoptosis and cell–cell-interaction [40]. Decreased expression of miR-206 observed in progressive disease correlated with the upregulation of target mRNAs related to inflammatory pathways, such as chemokine (C–C motif) ligand 19 (CCL19), C–X–C motif chemokine ligand 1 (CXCL1), interferon alpha and beta receptor subunit 2 (IFNAR2), NCK adaptor protein 2 (NCK2), protein tyrosine kinase 2 beta (PTK2B), protein tyrosine phosphatase, receptor type C (PTPRC), RAS guanyl nucleotide-releasing protein 1 (RASGRP), and tumor necrosis factor receptor superfamily member 25 (TNFRSF25). In addition, the biopsies from patients with progressive disease demonstrated reduced expression of miR-532-3p as well as elevated expression of target transcripts involved in apoptosis pathways including mitogen-activated protein kinase kinase kinase 14 (MAP3K14), tumor necrosis factor receptor superfamily member (TNFRSF10B)/TNF-related apoptosis-inducing ligand receptor 2 (TRAIL-R2), TNFR1-associated death domain protein (TRADD), and TNF-associated factor 2 (TRAF2). The authors suggested that concentration of aforementioned miRNAs and target mRNAs could correlate to clinical parameters and histological damage indices [40]. In turn, altered miR-21 regulations have been demonstrated by various researchers in acute and chronic models of kidney injury [47, 48].

The development of renal fibrosis is a final common pathway in the course of chronic kidney disease. It can be defined as excessive accumulation of extracellular matrix which in consequence leads to end-stage renal failure [38]. Exact sequence of molecular events resulting in kidney fibrosis remains unraveled, however, scientific data point to TGF‑β as the master regulator of this process which acts as the major driver of matrix degradation inhibition and matrix synthesis as well as myofibroblast activation [49, 50]. TGF‐β1 was found to regulate many miRNAs during the progression of diabetic kidney disease, but also its expression can be regulated by miRNAs. Epithelial to mesenchymal cells transition (EMT) may be an important factor contributing to renal fibrosis process and leading to subsequent structural destruction and renal failure [26]. Following kidney injury, stimulated resident fibroblasts undergo phenotypic transition to secrete a large amount of extracellular matrix (ECM) components which leads to constant excessive deposition of ECM proteins and in consequence to the loss of kidney function as a result of the destruction of kidney tissue [6, 51–53]. Some recent studies have criticized the role of EMT in renal fibrosis, however, numerous researchers believe that the extent of EMT process contribution to kidney fibrosis is probably disease-specific and context-dependent [54–56]. According to some studies, EMT process is regulated by miRNA, including miR‐200 family and miR‐205 [57, 58]. Li et al. [59] suggested that TGF-β stimulated renal fibrosis by inducing the expression of renal miR-433. In turn, Gomez et al. [60] and Lai et al. [61] suggest that the overexpression of miR-21, which is involved in different biological processes, such as proliferation, cell differentiation and apoptosis, plays a critical role in the progression of kidney fibrosis. miR-21 has been demonstrated to mediate epithelial disease progression in response to injury and also the development of fibrosis via the regulation of a metabolic switch [62]. TGF-β/Smad pathway was proposed to be the mechanism promoting increased miR-21 expression in fibrotic tissues. Moreover, Sun et al. [6] implied that miR-21 was a main driving force of fibroblast activation in various progressive diseases. High expression level of fibroblasts is associated with the formation of a double negative autoregulatory loop by miR-21, PDCD4 and AP-1 [6] Thesis concerning the involvement of miR-21 in this pathological process was confirmed by the observation that the therapy with antagomir-21 attenuated renal fibrosis. Therefore, the targeting this abnormally triggered feedback loop may provide useful in the treatment of fibrotic kidneys [38]. In addition, Zhong et al. [63] confirmed the role of miR-21 in renal fibrosis as they indicated that the suppression of miR-21 limited this process in rodent kidney disease models. Similarly, Gomez et al. [64] indicated a strong protection of kidneys against the development of fibrosis in miR-21^ − / −^ mice. miR-21 has been demonstrated to promote renal fibrosis by targeting PPARα and Mpv171 via the silencing of lipid metabolic pathway and aggravating ROS generation, respectively [48, 65]. Moreover, miR-21 silencing was found to boost PPARα/retinoid X receptor and the downstream pathways which protected mitochondrial function and lowered inflammation and fibrogenesis in renal tubule and glomeruli [60]. Microarray profiling of renal biopsy specimens obtained from three different pathological types of CKD patients enabled the identification of 40 miRNAs which were upregulated and 76 miRNAs downregulated in CKD renal tissues [56]. Yu et al. [56] by comparing 3 types of renal diseases with different degrees of fibrosis identified miRNAs profile which could be probably related to the progression of kidney fibrotic process. The authors suggested that two novel miRNAs, hsa-miR-3607-3p and hsa-miR-4709-3p might be engaged in the CKD-related fibrosis process. hsa-miR-4709-3p promoted while hsa-miR-3607-3p inhibited actin fibers assembling and cell motility [56]. In literature, functional roles of these two miRNA in human diseases are poorly characterized. On the basis of pathway enrichment analysis Yu et al. [56] implied that the above mentioned miRNAs might be involved in the regulation of actin cytoskeleton and thus contribute to the kidney fibrosis via targeting ITGB8 (belonging to the integrin beta chain family and encoding integrin αvβ8) and CALM3 (encoding calmodulin-3, a core intermediate calcium sensor in calcium signaling pathway) [56, 66]. The up-regulation of ITGB8 possibly leading to sustained activation of TGF-β signaling would be anticipated to occur in renal fibrosis. In addition, numerous studies have confirmed the significance of alterations in the concentration of intracellular calcium in cellular morphology changes and actin dynamic during EMT [56, 67]. Yu et al. [56] found that the most significant enriched pathway for hsa-miR-4709-3p was mTOR signaling pathway [56, 68]. Moreover, the other results of their study are in agreement with various reports pointing to miR-21, miR-29, and miR-200 family as key regulators in renal fibrosis [69–71].

The results of animal studies (UUO and the unilateral ischemia reperfusion injury (IRI) models) demonstrated that the lack or the silencing of miR-21-5p in both models amended albuminuria, kidney fibrosis and injury [48]. In addition, miR-21-5p-knockdown ceased the progression of fibrosis [101] which may imply a new therapeutic target for the treatment of kidney fibrosis [69, 72].

In turn, miR-29, which exerts anti-fibrotic effect, was found to be downregulated in focal segmental glomerulosclerosis (FSGS) and diabetic nephropathy (DN) group compared to control subjects [56]. Some studies have found that miR-29 family members play a role in the development of non-diabetic CKD. In animal models of CKD reduced miR-29 expression was related with disease development [72–74].

The protection of kidneys from fibrosis by hampering the deposition of ECM and preventing epithelial to mesenchymal transition (EMT) was also found to be associated with TGF‐β1-inhibited miRNA‐200a and miRNA‐141, respectively [75]. In addition, hsa-miR-200c-3p was demonstrated to be considerably diminished in all three types of CKD, while hsa-miR-200a-3p was hampered only in FSGS group compared to control. These results confirm the inhibitory impact of miR-200 family on EMT in the process of renal fibrosis initiation.

Hypertensive nephrosclerosis is another cause of chronic kidney disease and the most common cause of end-stage renal disease [43]. Wang et al. [43] demonstrated significantly higher expression of miR-200a, miR-200b, miR-141, miR-429, miR-205, and miR-192 in patients with hypertensive nephrosclerosis compared to that of normal controls. Moreover, they observed that the degree of upregulation correlated with disease severity and they found strong correlations between miRNA and proteinuria and GFR. Their finding implies the presence of the dose–response association between intrarenal miRNA expression and the severity of hypertensive nephrosclerosis. These results suggested that these miRNA might be of key importance in the pathogenesis of hypertensive nephrosclerosis and further end-stage renal disease.

According to studies, in most cases, miRNA levels decrease as CKD progresses, however, the exact mechanism of this reduction is unclear [33]. Chen et al. [8] demonstrated that in patients with CKD stages 3-5D, miR-125b, miR-145 and miR-155 levels were lower than in patients with normal kidney function. However, in their study, U6 was used as reference, which is not much reliable control of circulating miRNAs since the level of this small RNA (a part of the splicing complex) fluctuates broadly in human serum [76]. In addition, Neal et al. [33] found diminished levels of some circulating miRNAs in the serum of CKD patients, but again, in that study no reference gene was used in qPCR analysis of miRNA expression, thus the interpretation of their results is difficult. The analysis of miRNA expression in kidney biopsies revealed that the level of miR-223 was higher in biopsies of patients suffering from progressive chronic renal failure than in those of patients with stable CKD, which suggest a role of this miRNA in the aggravation of renal dysfunction [77]. miR-223 is considered to be an inflammatory miRNA and its possible role as a good biomarker of chronic micro-inflammation involved in the initiation and propagation of CKD has been suggested [78]. The expression of this miR-223 is modulated by the uremic toxin inorganic phosphate (Pi) and it was demonstrated to be increased in vivo in the aorta of CKD mice [9, 10, 79]. Moreover, miR-223 is an important regulator of hematopoietic system [80]. According to some studies, the alteration of miR-223 was associated with vascular calcification and low systemic miR-223 levels were associated with increased calcification and CKD stages [9, 78]. Metzinger-Le Meuth V et al. [62] implied that miR-223 and miR-155 (involved in osteoblastogenesis and osteoclastogenesis as well as inflammation) are essential in both bone and vessel regulation in CKD and that the deregulation of their expression could be responsible for the imbalance observed in CKD–MBD patients. Numerous studies also demonstrated the involvement of mir-155 in atherosclerosis, inflammation, cell proliferation oxidative stress related triggered by vascular injury [81, 82]. In turn, miR-223 has been reported to mediate switch of VSMCs towards the pro-calcification phenotype [10]. Fourdinier et al. [83] examined the expression of circulating miR-126 and miR-223 in the serum of 601 CKD patients (stages 1–5) followed-up for 6 years and 31 healthy controls and they found reduced miRNA levels in comparison with healthy controls. miRNA expression tended to be lower with advancing stages of CKD, however, after the correction for estimated glomerular filtration rate no association with loss of renal function was seen. In addition, Ulbing et al. [84] found a significantly lower systemic expression of miR-223-3p and miR-93-5p in patients with more advanced stages of CKD. Levels of these two miRNA significantly correlated with CKD stages, parameters of inflammation and kidney function, as well as indices of glucose metabolism. In turn, Fujii et al. [85] demonstrated also a negative correlation between miR-223 and eGFR as well as positive association between higher levels of circulating miR-197 and better kidney function. In the study of Japanese survivors of the earthquake, miR-126, miR-197, and miR-223 were found to be significantly associated with CKD [85]. According to studies, endogenous miR-126 may affect various vascular functions, such as angiogenesis, inflammation and leukocyte adhesion in atherosclerotic lesions and also endothelial dysfunction [86, 87]. It increases the expression of sirtuin1 and superoxide dismutase-2 reducing oxidative stress in ECs [88]. SIRT1 has strong profound antioxidative and anti-inflammatory properties and growing body of evidences indicate that the increase in its expression and/or activation exerts beneficial effects on ECs as a result of decrease in oxidative stress, prevention of endothelial senescence, enhancement of eNOS-derived NO bioavailability, and the promotion of mitochondrial biogenesis [89, 90]. SIRT1 was also found to be critical for the expression of SOD-2 gene which encodes manganese superoxide dismutase (MnSOD) protein, a powerful mitochondrial antioxidant enzyme that detoxifies the free radical superoxide [88, 91, 92]. High levels of circulating miR-126 were suggested to be associated with the maintenance of vascular function and a lower risk of CKD.

In turn, Zhang et al. [93] demonstrated higher miR-155 in a small group of hemodialysis patients. Due to the fact that during hemodialysis circulating miRNAs are not eliminated, it was suggested that it is not the procedure itself, but kidney impairment is responsible for the alterations in miRNA expression [94]. Since most studies have indicated the decrease in miRNA levels in patients with advancing CKD and in relation to evidence of considerably higher level of circulating RNases in patients with impaired kidney function [95], Neal et al. [33] made an attempt to determine whether enhanced degradation of either circulating miRNAs or of circulating exosomes in the plasma of patients was related to RNases activity. Their study confirmed accelerated miRNA degradation in patients with severe kidney disease, however, they failed to provide the explanation of this phenomenon. Reduced exosomal protection of miRNAs cannot be responsible for low levels of circulating miRNAs in kidney failure as authors did not observe differences in exosome abundance or stability of miRNA in uremic vs. normal serum [33].

Finally, according to studies, urinary miRNAs are released by cells in the nephron and downstreamed in the urinary tract [15, 96]. miRNAs in the urinary tract may be contained in membrane-bound extracellular vesicles, such as microvesicles and exosomes [32]. The exosomes present in urine are also considered to be a rich source of intracellular kidney injury biomarkers [97, 98]. It is anticipated that miRNA‐containing exosomes in the urine may provide valuable diagnostic and prognostic data for patients with kidney diseases [35]. Zang et al. [99] observed differential expression of urinary exosomal miR-21-5p and miR-30b-5p in individuals with diabetic kidney disease, however, until now, no urinary miRNA markers have been suggested for chronic kidney disease. Neal et al. [33] found no association between urinary level of miR-16, miR-21, miR-155 and miR-210 and kidney function. Only in case of miR-638, they observed a significant increase in its urinary levels in patients with stage 4 CKD in comparison to normal and stage 3 CKD patients (*p* = 0.006). On the basis of obtained results and data concerning urine excretion of miRNAs, they concluded that the kidneys seemed not to be involved in the physiological clearance of circulating miRNAs [33].

## miRNA and CKD-associated complications

CKD is considered to be an independent risk factor for cardiovascular disease (CVD). The accumulation of uremic toxins (such as indole-3-acetic acid (IAA), hippuric acid and p-cresylsulfate (pCS)) can damage vascular endothelial cells (ECs), leading to endothelial dysfunction manifested by the increase in proinflammatory cytokines and reduction in endothelial nitric oxide synthase (eNOS) [100, 101]. When the endothelium becomes dysfunctional, it acquires procoagulant and proinflammatory status and the production of NO is decreased [100, 102]. Endothelial dysfunction in CKD at least partly explains high prevalence of cardiovascular disease in this group of patients [62]. The search for miRNAs involved in this process brought the identification of some miRNAs which could be possibly play an important role. Abundantly expressed in ECs miR-126 has been suggested as a key factor due to the fact that it exerts pro-angiogenic effect and in consequence it stimulates blood vessel formation [103]. The release of this miRNA from ECs was found to be inhibited by atheroprotective laminar shear stress [104]. It has been also found that plasma concentration of miR-126 is diminished during the later stages of CKD and atherosclerosis. Moreover, the results of studies suggest that miR-126 can protect kidney tissue [105]. Endothelial miR-126 and miR-483 are considered to be important regulators of angiogenesis involved also in endothelial repair and homeostasis [106, 107]. Therefore, it seems that modulation of miR-126 expression may become a potential therapeutic method focused at the promotion of endothelial regeneration in vessels, as well as the protection of kidney tissue, therefore, limiting progression of atherosclerosis and damage due to CKD [62]. In contrast, miR-155 stimulates endothelial inflammation and mediates the development of atherosclerosis [108]. Recent studies have also indicated that miR-92a, which is induced by oxidative stress in ECs, participates in angiogenesis and atherosclerosis [109, 110]. miR-92a targets the 3′ untranslated region of mRNAs encoding sirtuin 1 (SIRT1), Krüppel-like factor 2 (KLF2), and KLF4 and via the inhibition of these molecules, it promotes the endothelial innate immune response and the subsequent vascular inflammation [101]. The results of miR-92a inhibition in animal model were associated with diminished endothelial inflammation and mitigated atherosclerosis [111]. Shang et al. [101] also indicated that elevated levels of miR-92a may exacerbate CKD and CVD through multiple mechanisms, including the worsening of renal function, impact on neointimal formation, and aggravation of endothelial dysfunction.

Adverse prognosis of CKD patients is related to endothelial dysfunction and arterial stiffness. Van Craenenbroeck et al. [112] demonstrated an association between worse renal function and higher plasma levels of inflammation-associated miR-146a. The expression of this miRNA can be stimulated by different proinflammatory stimuli, including IL-1, TNF-α, and Toll-like receptors (TLR) [113, 114]. It seems that the expression of miR-146a might be part of a mechanism responsible for hampering the excessive production of proinflammatory chemokines or cytokines in the course of inflammatory conditions, such as CKD [112].

Patients at more advanced stages of CKD have higher cardiovascular morbidity and mortality, which is related to the presence of atherosclerosis and/or vascular calcifications. Accelerated calcification process is a well-known consequence of uremia [15, 115]. Vascular calcification may stem from chronic kidney disease-mineral and bone disorder (CKD–MBD). This disorder is associated with the accumulation of phosphorus, elevated parathormone (PTH) and fibroblast growth factor 23 (FGF23) levels and vitamin D deficiency and leads to major complication of CKD [62, 116, 117]. The buildup of phosphorus, PTH, and FGF23 increases the risk of cardiovascular complications and mortality principally via development of vascular calcification (VC), endothelium dysfunction, and alterations of bone structure. Moreover, FGF23 stimulates left ventricular hypertrophy. Apart from enhancing cardiovascular risk, CKD–MBD impairs the quality of CKD patients’ life as a result of an increased risk of bone fractures and bone and joint complications [118]. The pathomechanism of vascular calcification in the course of CKD has been only partially elucidated. Imbalance of bone turnover observed in CKD patients exerts impact of transdifferentiation of VSMC into calcifying cells, endothelial dysfunction and osteoclastogenesis, leading to VC [62]. In response to various factors and signals, vascular smooth muscle cells (VSMCs) have been shown to be able to switch from ‘contractile’ (differentiated) phenotype into ‘synthetic’ (migratory and proliferative), the latter being associated with VC process observed in CKD–MBD [62]. miR-155 has been identified as another vital factor in CKD [119]. Both miR-155 and miR-223 are vital regulators of osteoclastogenesis [62]. Taibi et al. [62] found different expression of miR-126, miR-143, miR-145 and miR-223 in experimental murine model of moderate to advanced stages of CKD. miR-126 seemed to be involved in a phenotypic switch which directed the intima towards a deregulated state–endothelial dysfunction, while miR-143 and miR-145 played important roles in vascular disease [62, 87, 120]. According to studies, miR-143 impedes osteogenic differentiation, probably via targeting the Osterix transcription factor involved in VC, while miR-145 regulates osteoblastogenesis through the targeting of transcription factor Cbfb [121, 122].

In turn, Metzinger et al. [15] observed low serum level of miR-223 at high uremia, which implies that its modulation in CKD patients might be of value in handling of CKD-related complications. They also measured serum levels of miR-223 and miR-126 in a cohort of 628 patients (CKD stage 1–5 patients or on renal replacement therapy or healthy controls) in relation to all-cause mortality, and cardiovascular and renal events, and reported that serum levels of miR-223 and miR-126 in the following groups: CKD3B, CKD4, CKD5 and CKD5D groups were significantly lower compared to healthy controls [5]. Patients in whom miR-223 and/or miR-126 levels were below-median had slightly worse survival rate. However, miR-223 and miR-126 failed to be a prognostic marker of all-cause mortality, cardiovascular events or renal events [5]. In contrast, Fourdinier et al. [83] study of circulating miR-126 and miR-223 performed to assess the relationship between these miRNAs and cardiovascular and all-cause mortality revealed no association between both miRNA and mortality, cardiovascular disease and renal-related events.

## miRNA and the risk of graft rejection

Renal transplantation is the surgical procedure of choice for patients suffering from end‐stage renal disease since it is associated with greater survival and better quality of life in comparison to maintenance dialysis [35]. However, acute rejection and chronic allograft nephropathy still remain chief challenges despite the advancement in the field of immunosuppression. The monitoring of graft function with the use of invasive biopsies is risky and painful to transplant patients, and therefore, the finding of non-invasive and accurate biomarkers of allograft rejection and transplant failure is highly awaited by clinicians. Better understanding of underlying mechanisms and the identification of biomarkers which will enable early diagnosis of rejection and the risk of complications will be of great value. Precise and early diagnoses and the introduction of effective treatments of acute rejection will diminish mortality rates of renal transplant patients. Immune rejection of organ transplants pose a life-threatening complication and the fact that miRNAs can regulate the expression of genes involved in adaptive immunity have inspired researchers to explore this area of interest [77]. However, until now, the potential of miRNA in the field of renal transplantation has been analyzed just in some studies. It seems that miRNA expression patterns may serve as biomarkers of human renal allograft status. Anglicheau et al. [77] studied the expression of 365 mature human miRNAs in renal allograft biopsies (patients with acute rejection and those with normal allograft biopsy). They demonstrated that intragraft levels of miRNAs could be used to predict, with a high precision, acute rejection and renal allograft function. Intragraft levels of miR-142–5p, -155, -223, -10b, -30a-3p, and let-7c predicted renal graft function and the strongest association with graft function was observed in case of miR-142–5p and miR-10b. Several miRNAs highly expressed in acute rejection biopsies have been elsewhere shown to be involved in innate and adaptive immunity [123, 124]. Moreover, it was found that miRNA which were overexpressed in acute rejection biopsies, including miR-142-5p, -155, and -223, were also highly abundant in human peripheral blood mononuclear cells (PBMCs). The sensitivity and specificity of intragraft levels for the prediction of acute graft rejection was impressive (100% sensitivity and 95% specificity in case of miR‐142‐5p and 100% sensitivity and 95% specificity in case of miR‐155). Anglicheau et al. [77] reported considerable relationship between intragraft levels of overexpressed miRNAs and mRNA for T cell CD3 and B cell CD20. In their study, acute rejection biopsies were also characterized by under-expression of miRNAs (53 differentially expressed miRNAs, 43 miRNAs underexpressed, including let-7a and let-7c and only 10 were overexpressed) as compared to allografts with normal biopsy results. Finally, they suggested that altered expression of miRNAs during acute rejection may be associated with relative proportions of graft-infiltrating immune cells and resident renal parenchymal cells [77]. In another study, Sui et al. [125] found 20 differentially expressed miRNAs (12 downregulated, 8 upregulated) in acute rejection biopsies in comparison to normal allograft biopsies. Several studies indicated that miR-21 is upregulated during acute rejection [126], while Glowacki et al. [127] proposed that miR-21 can be a novel, predictive and reliable blood marker of kidney allograft fibrosis. According to Metzinger et al. [15], kidney-specific miRNA-146a is an interesting factor participating in the onset of graft rejection as it is also involved in ischemia–reperfusion injury [128] and its enhanced expression in dendritic cells was demonstrated to promote allogeneic kidney graft survival [129].

This thesis was confirmed by Igaz et al. [130] who found that corticosteroids decrease serum expression of some miRNAs. However, due to the fact that dexamethasone affects the levels of some, but not all miRNAs, it is plausible that some other mechanisms are involved. In other study [84], significant differences for miR-223-3p and miR-93-5p expression were found between CKD and kidney transplant patients, with higher levels in the latter group, which may mean that regulation of the amount of these two circulating miRNA is independent of kidney function. To limit the impact of renal impairment on miRNA expression, the authors analyzed CKD and KT patients with a similar eGFR. The expression of both miRNAs tended to normalize after kidney transplantation, and it was even higher in these patients than in healthy controls.

## Future opportunities related to miRNA use

The involvement of miRNAs in the pathophysiology of human diseases has launched great interest in their diagnostic and therapeutic opportunities. Manipulations of miRNAs can simultaneously influence various components of signaling pathway. Specific techniques of miRNA activity inhibition have been developed (i.e., antisense strategies, antagomirs, Decoy or Sponge technologies) [131–133]. It is plausible that the silencing of miRNAs involved in albuminuria, extracellular matrix accumulation, EMT and podocyte dysfunction or restoring miRNA function in kidney diseases in which miRNAs are downregulated may represent a potential therapeutic strategy [35]. It has been suggested that the modulation of dysregulated miRNAs in vivo may mitigate the manifestation of these diseases [6]. However, before it will be possible to successfully use new treatment strategies, there are many questions concerning the biology of miRNA which await answering. First, the regulation of miRNA synthesis is still not completely understood, for example the expression of miRNA which are located within introns of host genes is not the same as the expression of host gene [131, 134]. Moreover, there is a need to unravel all mechanisms of translational repression and activation and transcriptional effects as well as to identify miRNA specific targets [35]. Another challenge is associated with the development of safe and reliable systems enabling organ and cell‐specific delivery in a directed manner to avoid both off‐target adverse effects and the activation of the innate and adaptive immune response [35]. The preliminary results concerning the use of anti-miRs in animal studies are encouraging. For example, anti-miR-21 oligonucleotides have been shown to accumulate in the kidney and effectively inhibit miR-21 functions and that such blockade diminished macrophage infiltration in diseased kidneys [47, 131, 132]. In turn, anti-miR-192 therapy improved glomerular fibrosis in mouse models of diabetic nephropathy via a simultaneous reducing of collagen and fibronectin levels in the mesangial cells [38, 135].

It seems that the use of miRNA as reliable biomarkers for diagnosis, prognosis and response to therapy in renal diseases will outrun the introduction of miRNA-based therapies. However, also in this field there are some challenges. Until now, just a few studies analyzed miRNA profile in urine and blood as a potential biomarker for the detection of kidney injury and diseases. The understanding the pathophysiological role that given miRNAs play in the kidney is problematic since this organ comprises various types of cells in which the level of miRNA and the response to it may be diverse in different renal diseases [38]. Unfortunately, currently, our knowledge of miRNA-dependent regulation of normal and abnormal kidney function is not sufficient.

## Conclusions

miRNA have great potential of being sensitive and specific biomarkers in kidney diseases as they are tissue specific and stable in various biological materials. Studies on animal models, in vitro studies and human studies are constantly providing new data concerning miRNAs, however, a real challenge is to translate these experimental findings into reliable clinical diagnostic tools. Some promising non-invasive miRNA biomarkers have already been recognized in renal disease with the potential to enhance diagnostic accuracy, predict prognosis and monitor the course of disease as well as the response to treatment. Studies indicate that some miRNA levels can be increased in patients with CKD stages III–V and hemodialysis and decreased in renal transplant recipients (miR-143, miR-145 and miR-223) as well as elevated in patients with CKD stages III–V, decreased in hemodialysis patients and even more markedly decreased in renal transplant recipients (miR-126 and miR-155). However, large-scale clinical trials enrolling heterogeneous patients are required to evaluate the clinical value of miRNAs, confirm their accuracy and generalizability.
